# Personalized medicine: Striding from genes to medicines

**DOI:** 10.4103/2229-3485.71775

**Published:** 2010

**Authors:** Sunita R. Nair

**Affiliations:** *Cardiff Research Consortium (Knowledge Services), Capita India*

**Keywords:** Adverse events, drug discovery, personalized medicine, pharmacogenomics, therapy

## Abstract

Personalized medicine has the potential of revolutionizing patient care. This treatment modality prescribes therapies specific to individual patients based on pharmacogenetic and pharmacogenomic information. The mapping of the human genome has been an important milestone in understanding the interindividual differences in response to therapy. These differences are attributed to genotypic differences, with consequent phenotypic expression. It is important to note that targeted therapies should ideally be accompanied by a diagnostic marker. However, most efforts are being directed toward developing both these separately; the former by pharmaceutical companies and the later by diagnostic companies. Further, this companion strategy will be successful only when the biomarkers assayed are differentiated on a value-based approach rather than a cost-based approach, especially in countries that reimburse disease management costs. The advantages of using personalized therapies are manifold: targeted patient population; avoidance of drug-related toxicities and optimization of costs in nonresponder patients; reduction in drug development costs, and fewer patients to be tested in clinical trials. The success of personalized therapy in future will depend on a better understanding of pharmacogenomics and the extension of these scientific advances to all countries.

## INTRODUCTION

In medical practice of yore, caring and curing of patients had always involved patients’ family history, social background, behavior, and environment to tailor individual treatment. Physicians used this practice regularly but there was no consensus on the right approach.

The last 50 years have witnessed a revolution in medical practice through evidence-based medicine and development of standards of care. Despite the availability of a broad spectrum of therapies for the management of different diseases, “cure” often remains elusive for patients. These therapies may fail to be curative and may result in substantial adverse effects in a subgroup of patients. The evidence for effectiveness is often based on clinical and epidemiological studies of large cohorts. Such large studies report average responses overlooking individual differences. It is well known that there are interindividual differences in the response to therapy. These may be attributable to underlying molecular mechanisms.[1]

Personalized medicine attempts to provide an objective basis for exploring these differences. Since the inception of medical practice, physicians have tried to provide personalized medicine to patients: employing diagnostic tools, imaging techniques or assessing symptoms to determine disease conditions, and prescribe appropriate treatment. However, a more precise basis for providing personalized medicine would be the use of genetic information from patients to predict the prognosis of disease, initiate preventive measures, or select the most appropriate therapy for that individual patient. In other words, it means delivering the right medicine to the right people at the right time.

## ROLE OF PHARMACOGENOMICS AND PHARMACOPROTEOMICS

Advances in the field of genomics and genetics have yielded a vast amount of information, with researchers testing the integration of these results with the genome and medical history of patients. The completion of the Human Genome Project [Box 1] has elicited interest in pharmacogenomics. The scientific discipline that deals with the ability to test for variations in genes and their expression through molecular diagnostics and then to treat with targeted drugs is called pharmacogenomics.[2] Pharmacogenomics is a recently emerged discipline resulting from a fusion of pharmacogenetics with genomics. Pharmacogenetics deals with the drug response as a function of genetic differences among individuals. Enabled by high-throughput technologies in DNA analysis, genomics introduces a further dimension to individualized predictive medicine. These technologies include single nucleotide polymorphism (SNP) genotyping, haplotyping, and gene expression studies by biochip or microarrays.

**Box 1**

The human genome was mapped 10 years ago. The three billion base pairs mapped have provided valuable information in predicting the risk for disease and for developing targeted therapies.

Genetic profiling of an individual with respect to prediction of disease risk and drug response will have an impact on understanding disease pathogenesis, and enable truly personalized therapy. This need for treating rightly has been espoused in the survey of studies on adverse drug effects in hospitalized patients, which states that adverse drug reactions may rank as the fifth leading cause of death.[3] Pharmacogenomics will thus play an important role in assessing the risk of disease, drug development, and selecting the type of drug and its optimal regimen.

Besides pharmacogenomics and pharmacogenetics, pharmacoproteomics has an important role in the development of personalized medicines. Pharma-coproteomics uses proteomic technologies in drug discovery and development, and is a more functional representation of individual variation than that provided by genotyping. Proteomic technology, such as protein chips, is expected to contribute increasingly in clinical diagnostics in the next few years.[4]

## GENETIC VARIATIONS IN DRUG RESPONSE

Inter-individual differences in drug response are genetic-based, resulting in differences in metabolic pathways of drug action and elimination.[5] Genetic differences in the absorption, distribution, metabolism, and excretion (ADME) of drugs result in different plasma concentrations and excretion profiles, resulting in lower efficacy and toxic effects.[6] Polymorphisms [Box 2] in phases I and II drug-metabolizing enzymes lead to variations in metabolic profile among individuals.[7] Furthermore, genetic variations in receptors and transporters also produce variations in drug response; drugs reacting with polymorphic receptors will have reduced affinity. The drug will, therefore, have reduced efficacy in individuals carrying this polymorphism. For example, *β*_2_-adrenergic receptor (*β*_2_AR) – agonists for this receptor are used in the treatment of asthma; genetic polymorphisms of *β*_2_AR will result in a subset of nonresponder phenotypes.

**Box 2**

Polymorphism is an inherited genetic variation found among individuals in a population

## OPPORTUNITIES WITH PERSONALIZED MEDICINE

### Drug development

The pharmaceutical industry has traditionally followed the “blockbuster model” – a quest for path-breaking drugs with immense sales opportunities. The biopharmaceutical companies marketed about 40 blockbuster drugs in 2002, with total sales of nearly $90 billion.[2] Not all these drugs were completely effective for the prescribed population. These limitations make it difficult to use such drugs optimally in clinical practice; an increasing tendency to prescribe on a trial and error basis. Moreover, these drugs need to be tested in large and expensive clinical trials. More than ever, pharmaceutical companies are facing the burden of reduced research and development pipelines, commercial pressures, and meeting medical needs.

Personalized therapy has the potential to overcome these shortcomings; the shorter development cycle will reduce the cost of drug development. In 2005, the US Food Drug Administration (FDA) created guidance[8] for drug developers to link biomarkers to therapies in clinical development [Table 1]. Given the importance of developing genetic tests for targeted therapies, the National Institutes of Health (NIH) and the FDA are finding ways to regularize these tests in a way that allows for innovation without compromising patient safety.[9]

**Table 1:** 40 voluntary genetic data submissions made to the US FDA between 2004 and 2007.^8^

Submission types
‘-omics’
Pharmacogenomics
Proteomics
Metabolomics
Therapeutic Areas
Alzheimer’s disease
Cancer
Cardiovascular diseases
Depression
Metabolic disorders
Rheumatoid arthritis
Sepsis
Technologies
Genotyping devices
Microarrays
2D Gels
Mass spectrometry
NMR

Preclinical and clinical issues discussed

**Figure 1:**
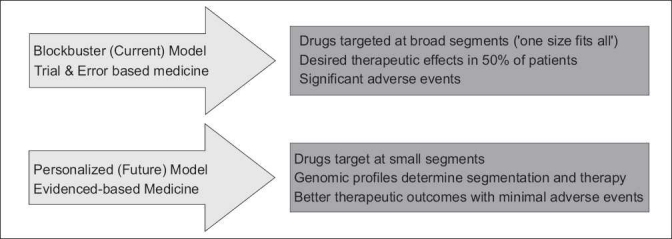
Comparison of drug development approaches

Biomarkers and surrogate markers can be used to identify responders and begin treatment earlier to reduce disease progression, help in adequate dose selection, exclude nonresponder patients, and thereby avoid toxicities in such patients. Furthermore, such predictive therapeutic modalities would reduce the financial impact of treatment for those patients who would not benefit [Figure 1].

### Diagnostic tests

Molecular diagnostics goes hand-in-hand with the development of personalized therapies. While there is interest to use pharmacogenomics in drug development, very few pharmaceutical companies evince interest in developing an accompanying diagnostic test. The difficulty in predicting clinical utility of biomarkers in drug development has resulted in pharmacogenomics being used with varying degrees of enthusiasm and investment. The majority of diagnostic test and therapy combinations are in oncology. Cancer is a complex and multifactorial disease that is life-threatening; treatments are expensive and associated with severe adverse events. The best known example being the HercepTest^®^ /trastuzumab (Herceptin^®^) combination from Dako and Genentech/Roche for the Her2-positive subset of breast cancer patients. Although diagnostic companies are focusing on diagnostic tests to predict success of therapies, the cost of using advanced techniques may become a limiting factor. This is especially so in Western countries where payers reimburse for diagnostics – the system evaluates innovative tests using a cost-based approach as against the value-based approach, wherein the costs are established relative to older tests using similar methodology. The value of using these diagnostic tests can be better appreciated by developing adequate clinical evidence. Limited knowledge on genotype–drug response–phenotype correlations is a barrier, delaying the use of pharmacogenomics into routine medical practice.[10,11] A systematic review of over 100,000 Medline-listed articles on pharmacogenomics reported that less than 2% of the studies were original research articles.[12]

#### Example of targeted therapy and companion diagnostics: Glivec™ (imatinib) and c-kit mutations

Gastrointestinal stromal tumors (GISTs) constitute the majority of gastrointestinal mesenchymal tumors, characterized by the expression of a proto-oncogene protein, CD117, which is detected by immunohistochemistry.[13] The identification of mutations in exon 11 and also exons 9 and 13 of the c-kit proto-oncogene coding for c-kit (CD117) in majority of GISTs resulted in a better understanding of their oncogenic mechanisms. Imatinib (Glivec™, Novartis) is a synthetic tyrosine kinase inhibitor, which is used in the management of interferon-resistant chronic myeloid leukemic (CML). Imatinib is also effective against a number of other tyrosine kinases including c-kit and platelet-derived growth factor (PDGF)[14] and is now considered as the drug of choice for metastatic and inoperable GIST. Until the discovery of imatinib mesylate, these tumor types were considered resistant to conventional chemotherapy. Imatinib is a good example of a drug targeted to a specific molecular defect of a tumor.

### Clinical trials

The application of pharmacogenomics will have an impact on the strategic design of clinical trials. Besides oncology, pharmacogenomic studies are also being conducted in other disease areas, such as cardiovascular diseases, conditions associated with the central nervous system, autoimmune disorders, and certain infectious diseases. Using genetic screening, potential responder populations can be identified before the enrolment process. This will help demonstrate drug efficacy in smaller sets of patients, without the risk of exposing nonresponders to adverse events. Genetic stratification of patients can enhance the statistical power of the study, thereby using smaller study populations and also reduce the overall time, resource use, and costs of the drug development process. Genotyping information can also help in finding and understanding “outliers” in laboratory findings or therapeutic profiles, attributed to genetic determinants.[15] Most of the newer therapies are tested across sites in multiple countries. The differential distribution of genetic variability in ethnic groups may limit the usability of drugs tested in one country to the other countries (especially those with different ethnic populations). Genotyping data from such ethnic groups will allow early identification of drug efficacy and toxicity, thus facilitating the conduct of smaller clinical trials in populations with similar genetic background.

## GENOMIC MEDICINE IN INDIA

While the developed countries are making great progress in the field of pharmacogenomics, developing countries such as India, Mexico, and South Africa are consolidating research in genomic medicine. The focus in these countries is to leverage the genetic variation in the population to explore linkages between genes, diseases, and environmental factors, and develop new therapies and diagnostic tests. In India, the concept of personalized therapy based on individual variations has existed since centuries through the practice of *Ayurveda*. India with its growing population and high rate of infectious diseases will find it extremely difficult to import expensive drugs and technologies for adequate healthcare management. The focus, therefore, is to look at biotechnology-based innovations. One such initiative is the Indian Genome Variation (IGV) Consortium,[16] a government-funded collaborative program among six laboratories of the Council of Scientific and Industrial Research (CSIR). This program aims to provide data on validated SNPs in over a thousand genes in 15,000 individuals drawn from Indian subpopulations; the IGV database has already been developed. The selection of the genes has been on the basis of their relevance as functional and positional candidates in many common diseases including genes relevant to pharmacogenomics. The important genetic information from this project is expected to facilitate research on disease predisposition, adverse drug reactions, and population migration.

## THE WAY FORWARD

The quest for genetic information has led to a better understanding of disease states and drug responses. Developing targeted therapies will not only reduce the overall cost of drug development, but will also ensure that appropriate therapies are given to patients on a more evidence-based approach. Among the ongoing initiatives of the Human Genome Project is the 1,000 genomes project, which is an effort by researchers from England, China, and the USA to sequence and compare the genomes of at least 1,000 people to study more precisely the variations linked to disease. The other is the Cancer Genome Atlas, an effort to collect more than 20,000 tissue samples from more than 20 cancers, and identify cascades of genetic changes that give rise to tumor growth. These initiatives will hopefully help use pharmocogenomics more routinely in medical practice in the coming decades.
